# Effect of Cu on the Formation of Reversed Austenite in Super Martensitic Stainless Steel

**DOI:** 10.3390/ma16031302

**Published:** 2023-02-03

**Authors:** Wen Jiang, Kunyu Zhao

**Affiliations:** 1 School of Chemistry and Chemical Engineering, Kunming University, Kunming 650214, China; 2 Department of Materials Science and Engineering, Kunming University of Science and Technology, Kunming 650093, China

**Keywords:** Cu, Ni, reversed austenite, super martensitic stainless steel

## Abstract

We investigated the effect of Cu on the formation of reversed austenite in super martensitic stainless steel by using X-ray diffraction (XRD), a transmission electron microscope (TEM) and an energy-dispersive spectrometer (EDS). Our results showed that the microstructure of the steels comprised tempered martensite and diffused reversed austenite after the steels were quenched at 1050 °C and tempered at 550–750 °C. The volume fraction of reversed austenite in the steel with 3 wt.% of Cu (3Cu) was more than that with 1.5 wt.% of Cu (1.5Cu). The transmission electron microscope results revealed that the reversed austenite in 1.5Cu steel mainly had the shape of a thin strip, while that in 3Cu steel had a block shape. The nucleation points and degree of Ni enrichment of reversed austenite in 3Cu steel were higher than those in 1.5Cu steel. The reversed austenite was more likely to grow in ε-Cu enriched regions. Therefore, Cu can promote reversed austenite nucleation and growth. The mechanical properties of 3 Cu steel are obviously better than those of 1.5Cu steel when tempered at 550–650 °C.

## 1. Introduction

Reversed austenite as an important tempered microstructure in iron and steel has been studied by researchers; the effects of alloying elements on the formation of reversed austenite have received considerable scholarly attention. Ni is the key element that promotes α-γ phase transition, while Ni gathers and provides the conditions for the reversed austenite nucleation [1,2,3,4,5,6]. In addition, N, Si, and other elements impact the phase transition process [7,8,9,10,11]. As an austenitizing element, the effect of Cu on the formation of reversed austenite is negligible.

Over the years, Cu has been used as an alloying element added to steels, and the main functions of Cu in steels are as follows. (1) Solid solution strengthening: After quenching the steel, Cu is dissolved in the steel matrix, which plays the role of solid solution strengthening. The solution-strengthening effect increases in proportion to the increase in carbon content. Cu in an austenitic matrix can be precipitated as finely dispersed Cu-rich phase particles during the aging process, which has an excellent dispersion-strengthening effect. Cu atoms segregated to stacking faults can also pin dislocations and induce a matrix-strengthening effect [12,13,14,15]. (2) Improvement of formability and processability of steels: Cu can improve the anisotropy of steel and reduce the work hardening index. (3) Improvement of corrosion resistance of steels: The enhanced corrosion resistance of steel is attributable to the formation of a dense copper layer on the surface of the steel during the corrosion process. However, some researchers believe it is related to anodic polarization. Studies have shown that only 0.1 wt.% of Cu can significantly improve the atmospheric corrosion resistance of steel [16,17,18,19]. To date, few studies have reported the effect of Cu on the formation of reversed austenite in super martensitic stainless steel. Thus, this study compared the solid solution and precipitation behavior and investigated their effect on microstructures and mechanical properties of Cu-free and Cu-containing super martensitic stainless steel [20,21,22]. By comparing super martensitic stainless steels with different Cu content, the effects of Cu on the formation of reversed austenite and mechanical properties were investigated.

## 2. Materials and Methods

Tested steel with extra low impurity contents was designed and melted in a vacuum-induction melting furnace (rated capacity: 25 kg, rated power: 100 kw, limited vacuum degree: 6 × 10^−3^ Pa, boosting rate: 0.05 Pa/min, maximum temperature: 1700 °C, cooling water pressure: 0.35 MPa). The ingots were hot forged into round bars with a 15 mm diameter. To obtain the complete lath martensite, the samples were quenched in oil at a temperature of 1050 °C for 0.5 h using a vertical-type furnace. Additionally, samples were tempered at temperatures of 550, 600, 650, 700, and 750 °C for 2 h. The chemical compositions of the test steels are shown in Table 1. An etching solution consisting of 1 g of FeCl_3_, 10 mL of hydrochloric acid and 120 mL of distilled water was used to etch the specimens for 10 s. The volume fraction of retained/reversed austenite at different temperatures was determined using PHILIPS APD-10 full-automatic X-ray diffraction instrument (XRD, Amsterdam, Netherlands) with Co radiation from 40 to 120° at a step interval of 0.02°. According to the integral intensity of diffraction peaks, the volume fraction of retained/reversed austenite was calculated by 6-line method and Formula (1), where *K* = *I*_0*A*111_/*I*_0*F*110_, I_0A111_ and I_0F110_ are the integral strengths of pure austenite and pure ferrite, respectively.

(1) $$\phi_{A}=1/(1+K\frac{I_{F110}}{I_{A111}})$$

The morphology and distribution of austenite in the martensitic matrix and the element concentration distributions of the microstructure were investigated using FEI high resolution transmission electron microscope (HRTEM, Hillsboro, OR, USA) after jet polishing with thin foil in a solution of 6% perchloric acid and 94% anhydrous ethanol.

## 3. Results

### 3.1. Effect of Cu on the Quenched Microstructure

**Figure 1:**
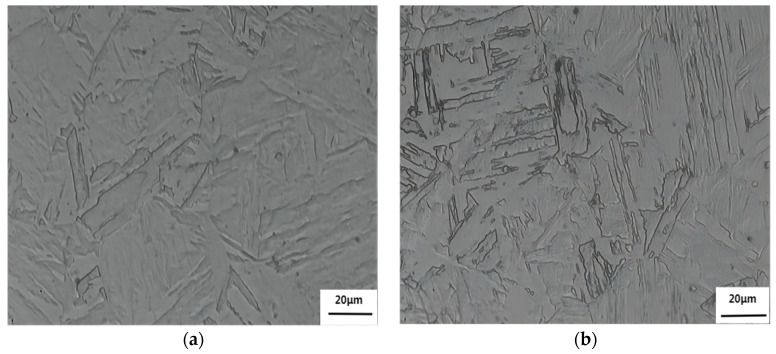
Microstructures of (**a**) 1.5Cu and (**b**) 3Cu steels quenched at 1050 °C.

**Figure 2:**
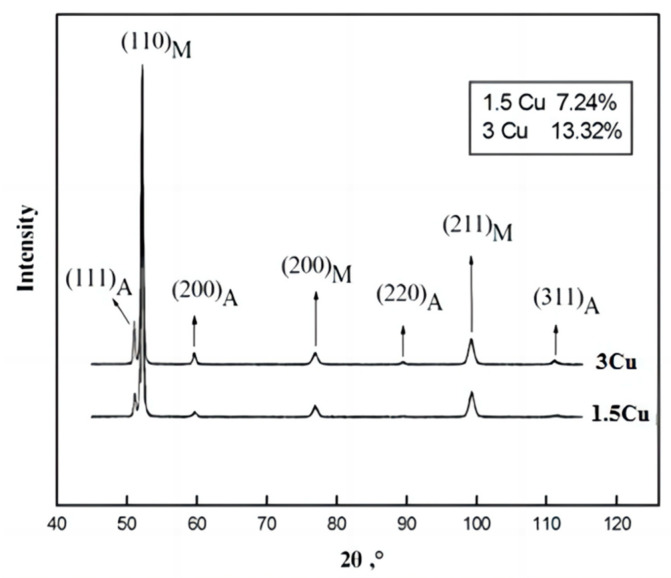
XRD patterns of retained austenite after quenching treatment of 1.5Cu and 3Cu steels. (M is martenite, and A is austenite).

At 1050 °C, the austenite grains of 1.5Cu and 3Cu test steels have reached uniformity and there is no excessive grain phenomenon. The microstructures of the two steels are both lath martensite at a quenching temperature of 1050 °C (Figure 1). There are several martensite lath bundles with different orientations in an original austenite grain. The volume fraction of austenite was measured with XRD to confirm the presence of austenite in the quenched microstructure of the two test steels. Figure 2 shows that the retained austenite is found in two steels. All the austenite diffraction peaks in the XRD patterns of 3Cu were stronger than those of 1.5Cu. After the volume fraction of austenite was measured, the volume fraction of retained austenite in 3Cu steel after quenching was 13.32%, while that in 1.5Cu steel was 7.24%. This result is because Cu will be completely dissolved in austenite when the steels are quenched at a higher temperature of 1050 °C. With the increase in Cu content, the dissolved Cu content in the austenite increased. Cu stabilized austenite as an austenite-forming element. Thus, the martensite transformation was delayed, and the volume fraction of retained austenite increased. In addition, Mn- or Si-alloying elements in the steel enhanced retained austenite. Thus, more austenites were retained in 3Cu steel than in 1.5Cu steel.

### 3.2. Effect of Cu on the Tempered Microstructure

**Figure 3:**
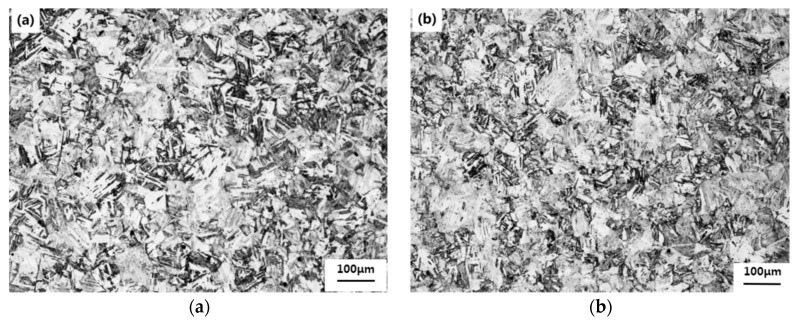
Microstructures of (**a**) 1.5Cu and (**b**) 3Cu tempered at 650 °C for 2 h after quenching at 1050 °C.

Figure 3 shows the microstructure of 1.5Cu and 3Cu tempered at 650 °C for 2 h after quenching at 1050 °C. The microstructure of the two test steels changed from thick lath martensite to fine-tempered martensite. After the test steels were tempered, the saturation of the supersaturated α-Fe decreased continuously due to the decomposition of the thick lath martensite and the precipitation of the carbides and intermetallic compound. At the same time, a few retained austenites were decomposed to form supersaturated ferrite and carbides, which were the tempered martensites.

**Figure 4:**
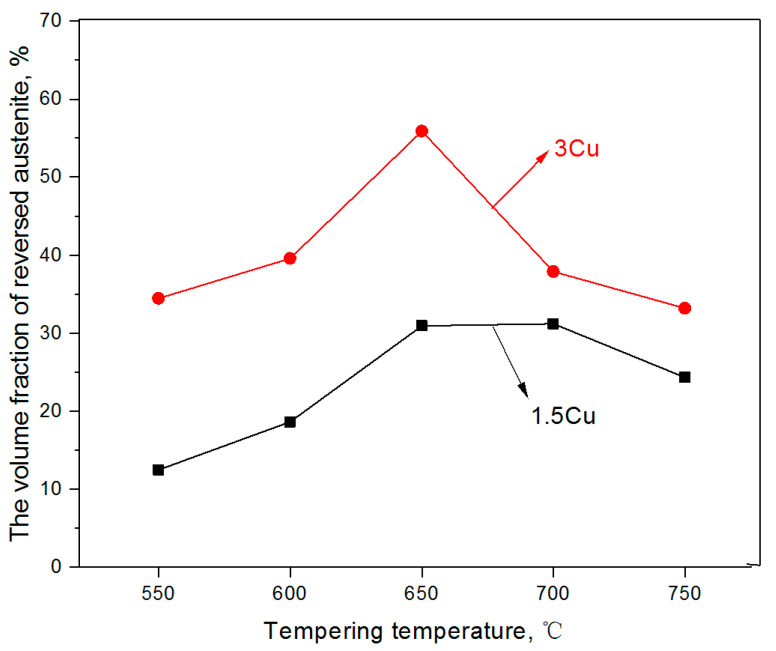
The curve of the volume fraction of reversed austenite and tempering temperature.

In addition, another type of austenite microstructure, namely, reserved austenite, was discovered in the two test steels. Reserved austenite was produced during the reversion of α to γ phase in the martensitic matrix. The variation of volume fractions of reversed austenite at different temperatures between 550 and 750 °C after quenching at 1050 °C are shown in Table 2 and Figure 4. A similar trend was observed in the amount of reserved austenite after the two steels were tempered. The volume fraction of reserved austenite of the two steels first increased and then decreased with the increasing tempering temperature and then reached a maximum value at 650–700 °C. The maximum values of the reserved austenite content of the two steels were 31.19% and 55.9%, respectively. Ni and Cu as the forming elements of austenite, can, in addition, increase the formation rate and decrease the formation temperature of reversed austenite. This is probably the main reason for the maximum amount of reversed austenite in 3Cu at 650 °C. Subsequently, the volume fraction of reserved austenite decreased due to the transformation from γ to α during the cooling process [23]. At all tempering temperatures, the volume fraction in 3Cu steel was greater than that in 1.5Cu steel. The 3Cu had a larger volume fraction of reversed austenite steel because Cu is an austenite-forming element. As a result, the greater the content of Cu, the easier the formation of austenite. In addition, the segregation of Cu reduces the faulting energy of the grain boundary, which is conducive to the diffusion of Ni. Earlier studies [24,25] have shown that Ni diffusion contributes to the formation of reversed austenite and increases its volume fraction. According to the above analysis, the increase in Cu content can promote the diffusion of Ni and the formation of reversed austenite. Thus, Cu and Ni can facilitate the formation of reversed austenite.

### 3.3. Enrichment of Cu and Ni in Reversed Austenite

According to the above analysis, the volume fraction of reversed austenite was greater in 1.5Cu and 3Cu test steels at a tempering temperature of 650–700 °C. To study the enrichment degree of Cu and Ni in the reversed austenite, TEM-EDS was used to analyze elements in the several forming regions of the reversed austenite at a tempering temperature of 650 °C. The average concentrations of Ni and Cu in the reversed austenite and in the matrix of two steels are shown in Table 3. As shown in Table 3, the Ni elemental concentration in reversed austenite in the two test steels was about three times higher than that in the matrix, indicating that Ni was enriched in the reversed austenite in the two steels. Furthermore, the content of Cu in the reserved austenite was more than that in the matrix, meaning Cu was also enriched in reversed austenite.

**Figure 5:**
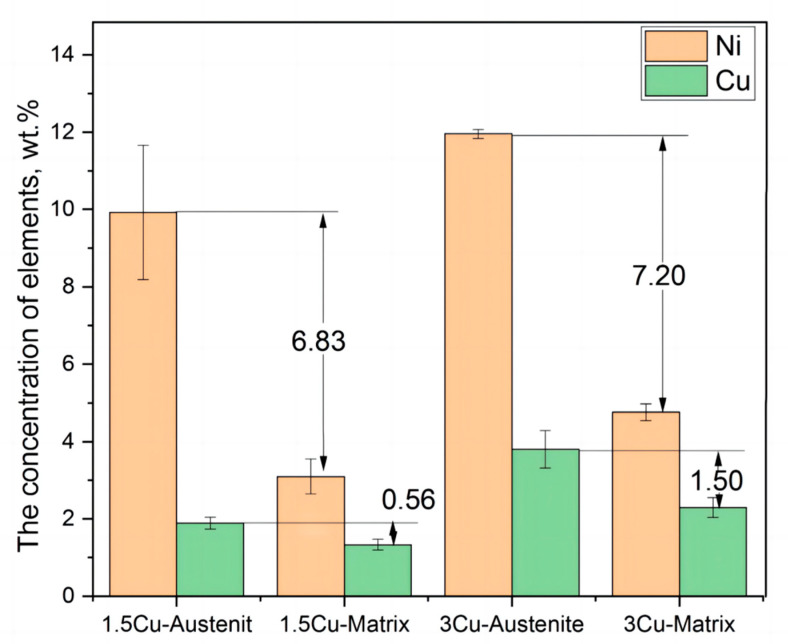
The concentration of Ni and Cu in reversed austenite and matrix in two test steels.

The data of Ni and Cu presented in Table 3 were represented in a bar chart (Figure 5). The concentration of Ni in 3Cu was higher than that in 1.5Cu. The average Ni concentration in 3Cu was approximately 11.96%, and the Ni concentration in 1.5Cu was approximately 9.93%. These results showed that increased Cu content could increase the enrichment concentration of Ni in reversed austenite, thus making the formation of reversed austenite easier in the Ni-enrichment area. In addition, from the bar chart of Cu element distribution shown in Figure 5, the difference in Cu concentration between reversed austenite and the matrix in 1.5Cu steel was 0.56%. However, the difference in Cu concentration between reversed austenite and the matrix in 3Cu steel was 1.5%. In other words, the difference in Cu concentration between reversed austenite and the matrix in 3Cu was two times greater than that in 1.5Cu steel, indicating that the enrichments of Ni and Cu in 3Cu steel were both higher than those in 1.5Cu steel. The enrichment of Cu was easier in the region with higher Ni concentration. As a result, the enrichment degree of Cu increased. Therefore, from the above analysis of the concentration of two elements, Cu and Ni contributed to the enrichment of each other in reversed austenite.

**Figure 6:**
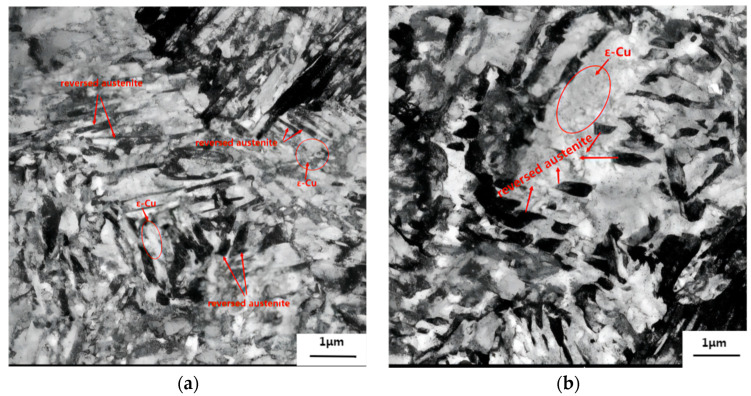
TEM bright field of (**a**) 1.5Cu and (**b**) 3Cu at tempering temperature of 650 °C.

To determine the effect of Cu on the reversed austenite formation, TEM was used to examine the microstructure of 1.5Cu and 3Cu test steels after they were quenched at 1050 °C for 30 min and tempered at 650 °C for 2 h. Figure 6 shows TEM bright field image. Figure 6a,b show dark long strips or block reversed austenite distributes in the boundary and interior of lath martensite. The reversed austenite in 1.5Cu steel was mainly thin and long along the boundary of martensite lath. However, the reversed austenite in 3Cu steel was blocked and was shorter and wider than that in 1.5Cu steel. The number of nucleation points of the reversed austenite of 3Cu steel was significantly more than that of 1.5Cu steel in the same photographic area. This phenomenon indicates that the addition of Cu in 3Cu steel can promote the diffusion of Ni and increase its enrichment degree and the number of Ni enrichment areas, thereby increasing the number of reversed austenite nucleation points. Therefore, the addition of Cu facilitated the formation of reversed austenite.

The different number of nucleation points was mainly attributed to the different shapes of reversed austenite in the two test steels. More nucleation points enhance the growth of reversed austenite grain to meet another growing austenite grain in the growth process. When the two grains meet, then the two grains will stop growing in the original direction and grow in other directions; thus, the shape of reversed austenite becomes a block. The nucleation number and shape of reversed austenite were relatively smaller and finer, respectively, with the increasing Cu content, which improved the mechanical properties of the test steel. As shown in Figure 6a,b, many fine precipitates were observed around the reversed austenite in the two test steels. These precipitates appeared in symmetrical or elliptical shapes, and the grain sizes of 20–50 nm were ε-Cu [12,26]. Compared with 1.5Cu steel, ε-Cu was distributed more densely around the reversed austenite of 3Cu steel (dashed region), indicating that the region with higher Ni enrichment increased the enrichment of Cu and promoted the precipitation of ε-Cu in this region. This phenomenon confirmed that the enrichment of Ni promotes the enrichment and precipitation of Cu in reversed austenite.

Given the above analysis of the two test steels with the same content of Ni, the volume fraction and nucleation points number of the reversed austenite in 3Cu steel are more than those in 1.5Cu steel, and the enrichment degree of Ni of the reversed austenite in 3Cu steel is greater than that in 1.5Cu, indicating that the addition of Cu promotes the enrichment of Ni. Moreover, Cu was also enriched around the reversed austenite or in the Ni enrichment area. The concentration of Cu and density of ε-Cu were also high in the area with a high concentration of Ni, indicating that Ni also contributed to the enrichment of Cu. Therefore, Cu and Ni synergically promote the formation of reversed austenite formation.

### 3.4. Effect of Cu on Mechanical Properties

**Figure 7:**
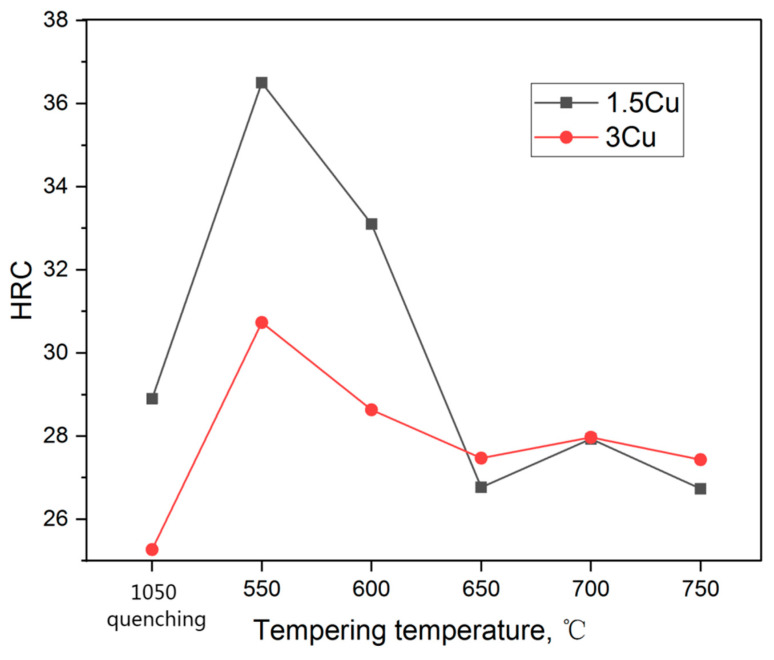
Variation of the hardness of the two test steels with tempering temperature.

Figure 7 shows the variation of Rockwell hardness (HRC) of 1.5Cu and 3Cu steels at different tempering temperatures. The HRC of the two test steels gradually decreased as the tempering temperature increased, until the latter reached the minimum at 650 °C, and then it slightly increased. During the tempering process, martensite desolated and transformed into reversed austenite with carbide precipitation. With increased tempering temperature, the volume fraction of reversed austenite gradually increased due to the increased driving force of α to γ transformation. Reversed austenite can reduce the hardness of steel as a softening phase [27]. Thus, HRC decreased at a temperature below 650 °C. With the increase in tempering temperature, the concentration of austenitic elements in the reversed austenite decreased gradually due to composition homogenization. The thermal stability of the reversed austenite decreased; as a result, the reversed austenite retransformed into martensite in the process of tempering and cooling, thereby decreasing the volume fraction of the reversed austenite. At the same time, the HRC of the test steels increased. Thus, the optimal tempering temperature for the reversed austenite and HRC of the two steels was 650 °C. The lowest HRC was obtained at 650 °C, consistent with the reversed austenite content test results.

**Figure 8:**
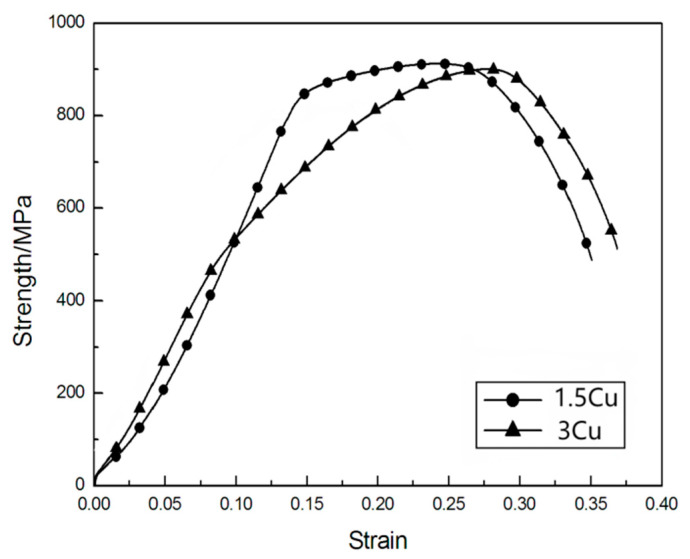
Strength-strain curves of the three tested steels tempered at 650 °C for 2 h.

The HRC of 3Cu is slightly smaller than that of 1.5Cu after quenching at 1050 °C, which is due to more retained austenite in the 3Cu steel. When the tempering temperature was between 550 °C and 650 °C, the HRC of 1.5Cu steel was significantly greater than that of 3Cu. However, no significant differences were observed in the HRC of the two test steels when the tempering temperature was above 650 °C. This phenomenon is because the 3Cu steel precipitated more fine grains and diffused a copper-rich phase during the tempering process to refine the grain at 550–650 °C. At the same time, there was the role of precipitation hardening [28,29]. Thus, the hardness can be improved to a certain extent. In addition, the increased volume fraction of reversed austenite in 3Cu significantly reduced the hardness [30,31], and the combined effect of the two factors reduced the HRC of 3Cu steel compared with that of 1.5Cu steel. At tempering temperatures above 650 °C, the refinement of ε-Cu relative to the grain weakened, along with the reduction in the reversed austenite, balancing each other and making the HRC of the two test steels remain basically the same. Figure 8 shows the strength-strain curves of the two tested steels tempered at 650 °C for 2 h. It can be seen that 3Cu steel has the highest tensile strength and the best plasticity. Although the yield strength of 3Cu steel is less than that of 1.5Cu steel, 3Cu has the largest strain hardening rate and the longest uniform deformation stage.

Table 4 shows the strength and elongation of 1.5Cu steel and 3Cu steel at tempering temperatures of 550–750 °C. As presented in Table 5, the tensile strength of 1.5Cu steel was 903.5–1008.8 MPa, the elongation was 16.66–19.62%, and the sectional shrinkage was 66.04–72.58%. The tensile strength, elongation, and sectional shrinkage of 3Cu steel were 902.5–985.2 MPa, 16.29–26.72%, and 61.51–73.17%, respectively. Overall, the elongation of 3Cu steel was larger than that of 1.5Cu, while the tensile strength of 3Cu steel was lower than that of 1.5Cu. It can be seen from the above data that the mechanical properties of 3Cu steel are obviously better than 1.5Cu steel when tempered at 550–650 °C. This phenomenon is because 3Cu steel has more reversed austenite after tempering.

**Figure 9:**
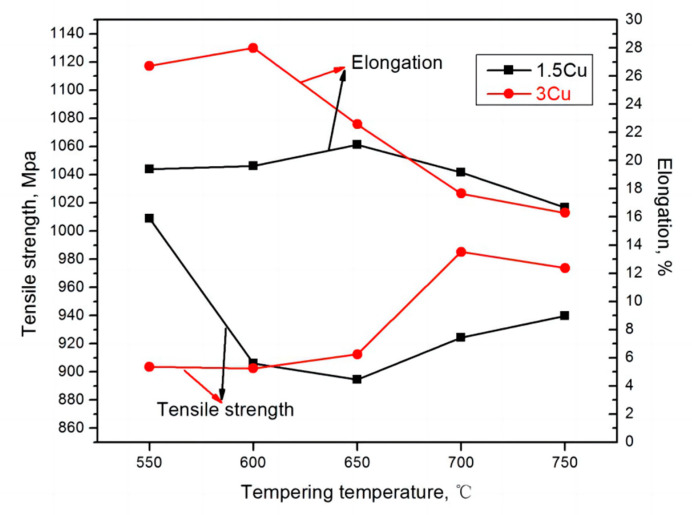
Variation of tensile strength and elongation of the two test steels with tempering temperature.

Figure 9 shows the curves of strength and elongation with tempering temperature. In general, the elongation of 3Cu steel is higher than that of 1.5Cu, while the tensile strength of 3Cu steel is lower than that of 1.5Cu. This is related to more austenite in the 3Cu steel after tempering. As the tempering temperature increased, the tensile strength of the two test steels at first decreased and then increased, whereas the elongation first increased and then decreased. When the tensile strength was minimum, the elongation became maximum. From the previous analysis, the test steel after tempering with the precipitation of reversed austenite. The volume fraction of reversed austenite with the increased tempering temperature first increased, gradually decreased, and peaked at 650 °C. The soft and tough phases and the reversed austenite were diffusely distributed in the martensitic laths and austenite grain boundaries after tempering, which could absorb the deformation work during plastic deformation of the material at room temperature. The plasticity induced by the martensitic phase transformation can significantly improve the plastic toughness of the material [32,33,34]. Therefore, the strength and elongation of the two test steels changed with the change in austenite content, indicating that the mechanical properties of the test steels were related to the reversed austenite. Reversed austenite has a dual effect on super martensitic properties. On the one hand, an appropriate amount of fine block reversed austenite has a certain strengthening effect and toughening effect, but too much reversed austenite will reduce the strength of steel.

**Figure 10:**
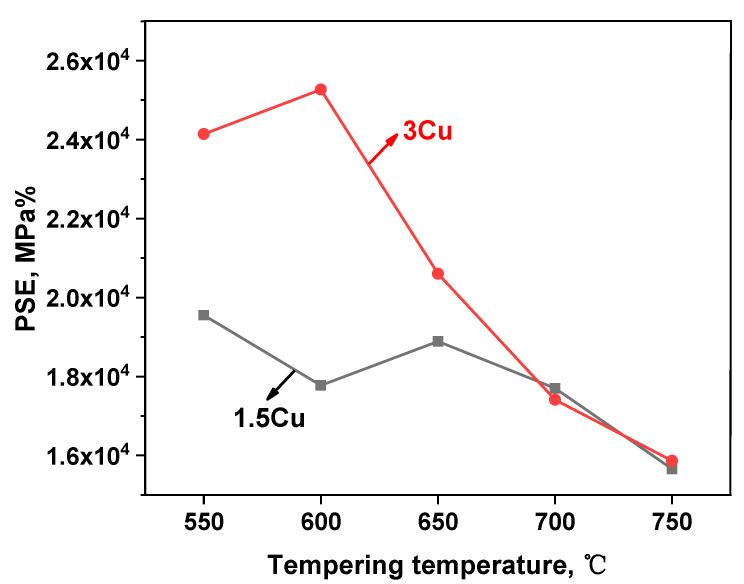
Variation of strength and elongation of the test steels with tempering temperature.

Because the strength and elongation of the two test steels varied, the strong plastic product (PSE) of the two test steels was calculated (Table 4) to illustrate their comprehensive mechanical properties. The curve graph of the PSE of the two steels is shown in Figure 10. The PSE of 3Cu steel was greater than that of 1.5Cu steel. This result showed that the strength and plasticity could be improved by adding Cu to some extent. The super martensitic stainless steel containing Cu had a high PSE value (15,657–25,270 MPa%). This result further indicated that the synergistic effect of Cu and Ni contributed to the formation of reversed austenite and improved the strength and toughness of the steels.

## 4. Discussion

Given the above analysis, Cu was enriched in the reversed austenite to a certain extent in 1.5Cu and 3Cu steels. The process of Cu enrichment can be explained using diffusion theory. The diffusion of Cu can be expressed as follows [35]:

*D* = *D*_0_ exp (−*Q*/*RT*) (2)

where *D* is diffusion coefficient (cm^2^/s), *D*_0_ is frequency factor (cm^2^/s), *Q* is activation energy (kJ/mol), *R* is the Boltzmann constant, and T is diffusion temperature (K). The diffusion coefficient of Cu in martensite (*D_Cu-α_*), Cu in austenite *(D_Cu-γ_*), and self-diffusing (*D_Cu-self_*) are calculated using the following formulas:

*D_Cu-α_* = 300exp (−284,000/*RT*) *D_Cu-γ_* = 0.19exp (−272,000/*RT*) 
*D_Cu-self_* = 0.78exp (−211,000/*RT*) (3)

**Figure 11:**
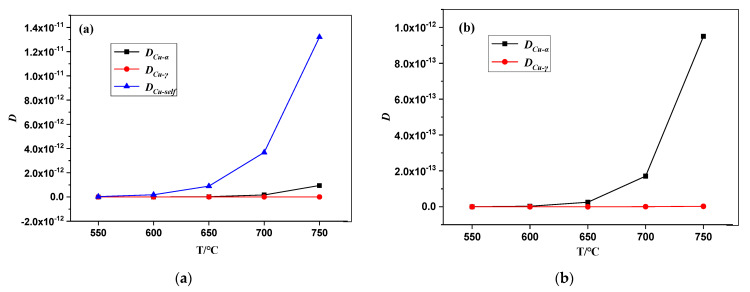
The curves between the diffusion coefficients and tempering temperature. (**a**) The curves of *D_Cu-self_*_,_ *D_Cu-α_*, and *D_Cu-γ_*; (**b**) The curves of *D_Cu-α_* and *D_Cu-γ_*.

Based on Formula (3), the data and curves of *D_Cu-α_*, *D_Cu-γ_*, and *D_Cu-self_* at different tempering temperatures are shown in Table 5 and Figure 10, respectively. As presented in Table 5, values of *D_Cu-self_* were larger than those of *D_Cu-α_* and *D_Cu-γ_* at all the tempering temperatures, and the largest value was about two to four orders of magnitude. *D_Cu-α_* was 1–2 orders of magnitude larger than *D_Cu-γ_*. The variations in values of the three diffusion coefficients were more significant with increasing tempering temperature. The relationship between the diffusion coefficients and tempering temperature is represented in Figure 11. In the tempering temperature range of 550–750 °C, the three diffusion coefficients exponentially increased in the following order: *D_Cu-self_ > D_Cu-α_ > D_Cu-γ_*. When the tempering temperature was in the range of 550–600 °C, *D_Cu-self_* was small and stable, while the self-diffusion rate of Cu was slow. When the tempering temperature was 600–750 °C, the self-diffusion rate of Cu became faster with the increasing temperature, and ε-Cu was gradually formed. Figure 11b is a larger version of Figure 11a, and it shows an increase in *D_Cu-α_* starts. Because *D_Cu-γ_* was relatively small, the Cu atoms in austenite could be seen as stationary compared with the rapid diffusion of Cu atoms in martensite. Then, when the Cu atoms in martensite entered the austenite, its diffusion rate rapidly decreased and became stationary. As the tempering temperature increased, more Cu atoms entered the austenite and enriched the reserved austenite. Therefore, the volume fraction of reversed austenite rapidly increased within the tempering temperature range. In addition, both Ni and Cu played a pivotal role in the enrichment of reversed austenite.

## 5. Conclusions

1. The matrices of 1.5Cu and 3Cu super martensitic stainless steels were quenched at 1050 °C. A small amount of retained austenite in two test steels and the addition of Cu increased the volume fraction of retained austenite after quenching.
2. The two test steels were quenched at 1050 °C and tempered at 550–750 °C. The tempered microstructures included tempered martensite, reversed austenite, and ε-Cu. The reversed austenite content in both test steels initially increased, then decreased, and finally reached a maximum at 650–700 °C as the tempering temperature increased. The reversed austenite content in 3Cu steel was more than that in 1.5Cu steel, and the distribution in the matrix was also denser.
3. The degree of Ni enrichment and the number of enrichment areas within the reversed austenite in 3Cu steel was higher than that in 1.5Cu steel. The enrichment of Cu elements was also more pronounced in the area of high Ni concentration in the reversed austenite, and more ε-Cu precipitated near this area, indicating that Cu contributed to the nucleation and growth of reversed austenite.
4. The addition of Cu to the test steels facilitated the reversed austenite formation, and the mechanical properties of 3Cu steel are obviously better than those of 1.5Cu steel when tempered at 550–650 °C.

## Figures and Tables

**Table 1:** Chemical composition (wt%) of the test steel.

Steel Grade	C	Mn	Si	Cr	Ni	Mo	W	Cu
1.5Cu	0.021	0.4	0.27	14.78	6.5	2.04	0.8	1.44
3Cu	0.02	0.38	0.26	14.78	6.6	2.05	0.88	2.74

**Table 2:** The volume fraction of austenite at different heat treatment.

Steel Grade	1050 °C Quenching	550 °C Tempering	600 °C Tempering	650 °C Tempering	700 °C Tempering	750 °C Tempering
1.5Cu	7.24	12.48	18.63	30.93	31.19	24.33
3Cu	13.32	34.46	39.55	55.90	37.87	33.18

**Table 3:** EDS analysis result of austenite and matrix at a tempering temperature of 650 °C.

Element (wt.%)	1.5Cu			3Cu		
	Austenite	Matrix	Δ	Austenite	Matrix	Δ
Ni	9.93	3.10	6.83	11.96	4.76	7.20
Cu	1.89	1.33	0.56	3.79	2.29	1.50

**Table 4:** The data of mechanical properties in 1.5Cu and 3Cu steels.

Cu Content (wt.%)	Tempering Temperature	Tensile Strength (MPa)	Elongation to Failure, εu (%)	Product of Strength and Elongation (MPa%)
1.5Cu	550	1008.82	19.38	19,550.93
	600	905.84	19.62	17,772.58
	650	894.35	21.12	18,888.67
	700	924.19	19.15	17,698.24
	750	939.81	16.66	15,657.23
3Cu	550	903.53	26.72	24,142.32
	600	902.48	28.00	25,269.44
	650	912.40	22.58	20,601.99
	700	985.25	17.67	17,409.37
	750	973.70	16.29	15,861.57

**Table 5:** The diffusion coefficients at different tempering temperatures.

T	*D_Cu-α_*	*D_Cu-γ_*	*D_Cu-self_*
550	2.85 × 10^−16^	1.04 × 10^−18^	3.18 × 10^−14^
600	3.07 × 10^−15^	1.01 × 10^−17^	1.86 × 10^−13^
650	2.55 × 10^−14^	7.72 × 10^−17^	8.97 × 10^−13^
700	1.71 × 10^−13^	4.77 × 10^−16^	3.68 × 10^−12^
750	9.50 × 10^−13^	2.47 × 10^−15^	1.32 × 10^−11^

## Data Availability

The data that support the findings of this study are available from the corresponding author, [Jiang W], upon reasonable request.
